# The Hospitals and Hospital Saturday

**Published:** 1892-05-07

**Authors:** 


### The Hospital

An Institutional Journal of

**Science, Medicine, Nursing, and Philanthropy.**

*For Hospitals, Asylums, Infirmaries, Dispensaries, Medical Practitioners, Students, Nurses, and the Charitable Public.*

Vol. XII.—No. 293.] [May 7, 1892.

### THE HOSPITALS AND HOSPITAL SATURDAY.

Silently and surely great changes have been effected in the constitution and spirit of the Metropolitan Hospital Saturday Fund. At the present time it has associated with it a staff of able officials who understand their business, and who labour devotedly and intelligently for the public good. We desire to direct the attention of metropolitan hospital committees to these facts, and at the same time to urge upon them the importance and duty of placing themselves in communication with Mr. Reginald B. Acland, the Chairman of the Council, with a view of ascertaining to what extent it may be possible to awaken and secure more adequate support for the hospitals from the workshops in the various districts in London. Two plans of securing this support have been adopted in the provinces. In Birmingham the plan is to levy a subscription of so much a week upon each of the employés in all the workshops co-operating with the Hospital Saturday Fund. Out of this money subscriptions are paid to the various hospitals in proportion to the amount of relief required by the subscribing employés, workmen, or artisans, their wives, and families; and on the eve of Hospital Saturday in each year all the money which the box then contains is sent as a free gift to the Central Fund. In the Potteries they have another system, which is well worthy of general consideration. All the works are in direct communication with the North Staffordshire Infirmary, and a system has been organised by which the Infirmary authorities issue to each workshop books containing letters of introduction, which the foremen fill in, as occasion may require, in favour of any of the employés or their families who may be ill. In this way the hospital is left free to give such relief, and such only, as each case may require, and both the hospitals and the workshops are enabled to ascertain the amount and the cost of the relief given to the artisans in each year. This measure of work done stimulates the contributions from the work-people, who believe in a *quid pro quo*. It further gives them an opportunity of making a free gift to the hospital, the amount of which at the North Staffordshire Infirmary reaches £500 per annum. At Birmingham, the working classes give £10,000 through the Hospital Saturday Fund, though they cannot claim the whole of this money as a free gift, seeing that no means have yet been adopted of ascertaining how many artisans go to the hospitals without tickets, and whether or not the contention of some of the medical staff is correct, that the Birmingham hospitals expend annually on the working classes much more than the whole £17,000 which they at present subscribe. If tickets were abolished and letters of introduction substituted, a little co-operation between workshops and hospitals would enable the precise facts to be arrived at. For this reason we prefer the scheme in force in the Potteries.

It was suggested, as will be seen from the report given in another column, at a conference of Hospital Saturday workers last week, that an attempt should be made to induce every workshop in the metropolis to establish a box and to put into it a weekly subscription from each of the employés, together with an adequate contribution from the masters. The money so obtained was to be used, in the first instance, to pay the fees of the local medical practitioner, to whom the workman or his family would be first referred by the foreman, and who would attend them in all ordinary illnesses. If it was necessary for a case to go to a hospital, because it required nursing and other advantages not obtainable at the patient's home, then a letter of introduction would be sent to the hospital with the patient from the workshop, which would secure the necessary further treatment there. Thus the interests of the artisans, the hospitals, and the general practitioners of medicine would be alike protected. The workshops would keep an account of the number of cases sent with letters, the hospitals would keep an account of the cost of treating the cases, and it would then be possible to see exactly what proportion the monies contributed by the workshops bore to the relief given to the workmen by the hospitals. The London working man is strong in his right to obtain from the hospitals what he requires when ill for an adequate payment; and such a system as this would enable those who are willing to pay for medical relief to see that their less thrifty fellows who declined to contribute to the boxes did not obtain free relief without their being aware of it.

It was also proposed that the hospitals should co-operate with the Hospital Saturday authorities in the districts which they each specially serve, and in this way it is believed that every workshop might easily be induced to join such a general organisation for the protection and benefit of the working classes of London. On the day fixed for Hospital Saturday in each year the boxes would be opened and their contents sent to the Saturday office as a free gift from the workshop. The total thus collected would be distributed without privileges or tickets amongst the various hospitals by the Council after setting aside a certain percentage to defray the cost of supplying medical and surgical appliances to those of the contributors who might need them. Each workshop would be further able to arrange with the various hospitals for direct representation by virtue of their contributions, although such contributions were in reality payment for relief afforded, because it would be reasonable for the institutions to give certain privileges to the artisans as a sort of bonus on their efforts to pay the cost of their treatment when ill. Mr. Webster, whose experience in the matter of workshop collections is almost unique, pointed out that it would be difficult to carry out such a scheme in London, because the workshops, as a rule, were so small. In the provinces it is not an unusual thing for a workshop box to produce £200 in the course of the year, whereas, in London the establishments are so small as to make it impossible to hope that, as a rule, any but relatively small sums could be so collected. This being so, it was proposed to modify the scheme by inducing all the workshops to send their contributions intact to the Hospital Saturday Office, and to leave the authorities there to make the arrangements with the hospitals, by which accurate accounts may be kept of the number of patients sent, the cost of such patients, and the contributions given towards defraying this expenditure in the aggregate. In other words, the Hospital Saturday collections in London would in future be divided into two parts: one part would represent the money sent to the hospitals to defray the cost of the cases which were admitted to treatment through the Hospital Saturday Fund, and the other part would be the balance contributed beyond this outlay, which would constitute the free gift of the working men of London.

It is most desirable, having regard especially to the enormous increase in the growth of the out-patient department, that steps should be taken to regulate the admissions, and to secure that as far as possible those patients who can afford to do so, shall contribute towards the cost of their treatment. For these reasons we venture to commend the proposals very cordially to the consideration of the hospital authorities, and we hope very many of them will put themselves in communication with Mr. Acland, the chairman of the Hospital Saturday Fund, with a view to the early adoption of this practical scheme. Its great merit is, that by giving the institutions, the Hospital Saturday Fund authorities, and the individual workshops an accurate knowledge of the actual cost of treating the artisan patients, it will be possible to stimulate the working-classes to exert themselves sufficiently to secure that their contributions shall bear something like an adequate proportion to the advantages they at present receive from the hospitals, which, it must always be remembered, were founded originally for the relief of those patients, and those only, who are unable when ill to pay for adequate medical treatment.

There remains the question of the payment of the general practitioner. Unfortunately, working-men are too prone to believe that, whatever they may suffer from, the best thing to do is to resort to some specialist, who is supposed to be able to deal with their cases in a way not within the means of an intelligent and capable general practitioner. Anything more opposed to common sense than this view it is difficult to conceive. If the Hospital Saturday Fund are prepared to educate their contributors to the advantage and importance of their first of all resorting to the local medical man when ill, they will have done much to promote the happiness of the home, and to secure the speedy recovery of the patient without unreasonable delay or risk. Workmen have it in their power to help themselves by securing, under this system, the best of medical treatment in their own homes at a reasonable cost, and to assist the hospitals by relieving them of the enormous number of trivial cases which ought never to present themselves for treatment at those institutions.

